# Optimal anti-platelet therapy for older patients with acute coronary syndrome: a network meta-analysis of randomized trials comprising 59,284 older patients

**DOI:** 10.1007/s11239-023-02875-x

**Published:** 2023-08-07

**Authors:** Shuang Zhou, Wenhui Li, Qian Xiang, Zhe Wang, Hanxu Zhang, Guangyan Mu, Zhiyan Liu, Yimin Cui

**Affiliations:** 1 Department of Pharmacy, Peking University First Hospital, No. 8 of Xishiku Street, Xicheng District, Beijing, 100034 China; 2 School of Pharmaceutical Sciences, Peking University Health Science Center, No. 38, Xueyuan Road, Haidian District, Beijing, 100191 China; 3 Institute of Clinical Pharmacology, Peking University, No. 38, Xueyuan Road, Haidian District, Beijing, 100191 China

**Keywords:** Anti-platelet therapy, Older patients, De-escalation, Acute coronary syndrome, Network meta-analysis

## Abstract

The aim of this study was to identify the optimal anti-platelet therapy in older acute coronary syndrome (ACS) patients with a mean age ≥ 60 years by comparing the efficacy and safety of different anti-platelet therapies. The selection of antiplatelet therapy in older patients with ACS is a clinical challenge. Numerous evidences indicate that the de-escalation of dual anti-platelet therapy (DAPT) or P2Y12 inhibitor monotherapy may reduce bleeding risk without increasing thrombotic events. However, there is a lack of systematic reviews and optimal strategy analysis regarding older ACS patients. Randomized controlled trials (RCTs) of anti-platelet therapy in older ACS patients were identified. Major adverse cardiovascular events (MACE) were the primary outcome. Secondary outcomes included all death, cardiovascular death, myocardial infarction, stroke, stent thrombosis, and trial-defined major bleeding. Frequentist and Bayesian network meta-analyses were conducted. Treatments were ranked on posterior probability. Summary odds ratios (ORs) were estimated using Bayesian network meta-analysis. A total of 12 RCTs including 59,284 older ACS patients treated with five anti-platelet strategies were included. Ticagrelor monotherapy after 3 months DAPT was comparable to the other strategies (OR 0.73; 95% CI 0.32–1.6) in terms of MACE risk. Additionally, P score analysis and SUCRA Bayesian analysis showed that it was the most beneficial treatment for all deaths, cardiovascular death and revascularization. For safety, although there was no significant difference in direct comparisons, both SUCRA Bayesian (0.806) and P score (0.519) analysis suggested that ticagrelor monotherapy was the safest strategy. The current evidence demonstrated that ticagrelor monotherapy after 3 months DAPT may be a promising approach for achieving a more favorable balance between risk and benefit for older ACS patients, with a relatively low bleeding risk and without an increased risk of MACE events. Moreover, it remains the preferred option for clinical outcomes such as all death, CV death and revascularization. Further high-quality and long-term studies are required to validate anti-platelet therapies among older ACS patients.

**Supplementary Information:**

The online version contains supplementary material available at 10.1007/s11239-023-02875-x.

## Introduction

Acute coronary syndromes (ACS) are a leading cause of morbidity and mortality in the world [1, 2]. Among the patients with ACS, the majority are elderly, with an average age of 65–70 years, and approximately 30% are over 75 years [3]. The challenges in the management of older patients with ACS were highlighted several years ago [4], and are becoming even more urgent with the increase in life expectancy and aging population worldwide [5]. However, older patients tend to be under-represented in clinical trials [6]. The atherosclerosis of older patients may be different, with more extensive coronary calcification and a higher likelihood of multivessel disease. Moreover, biological changes, including diminished renal clearance and lower muscle mass, lead to altered pharmacokinetics and pharmacodynamics [7].

Dual anti-platelet therapy (DAPT) is the key cornerstone in the management of patients with acute coronary syndrome (ACS) [8, 9]. The regimen and duration of DAPT vary according to clinical characteristics as well as ischemic and bleeding risk. In non-ST-elevation ACS (NSTE-ACS) patients aged ≥ 70 years, the incidence of bleeding in combination with ticagrelor (90 mg twice daily) and aspirin was 24% [10]. Anti-platelet is associated with increased bleeding risk in the older patients, as well as thromboembolism risk [5]. For different antiplatelet, the POPular AGE study [11], which included NSTE-ACS patients aged ≥ 70 years, showed no difference in the ischemic endpoint between the potent P2Y_12_ inhibitor group and the clopidogrel group, but demonstrated significantly higher bleeding risk in the potent P2Y_12_ inhibitor group. Similarly, results from a Swedish observational registry including patients older than 80 years demonstrated an increased incidence of mortality and bleeding in patients treated with ticagrelor compared with clopidogrel [12]. In summary, the optimal potency of anti-platelet therapy in these patients usually with both high ischemic and bleeding risks remains unclear [13]. There is no consensus on optimal anti-platelet regimens for older adults with ACS.

Previous network meta-analyses [14, 15] have demonstrated that de-escalation of DAPT is a highly effective approach for treating ACS, resulting in fewer bleeding events while not increasing the incidence of ischemic events. In addition, another meta-analysis [16] was conducted to identify the most appropriate P2Y_12_ inhibitors for older ACS patients. As a further step, we aimed to determine the optimal antiplatelet therapy strategy for older ACS patients through a comprehensive network meta-analysis that incorporated both direct and indirect comparisons to assess the efficacy and safety.

## Methods

### Search strategy and selection criteria

An electronic search of relevant literature was conducted using the PubMed, EMBASE, Clinical Trials, and Cochrane Library databases from inception to June 15, 2021. The search terms used were as follows: “acute coronary syndrome”, “ST segment elevation myocardial infarction”, “non ST segment elevation myocardial infarction”, “unstable angina”, “ticagrelor”, and “clopidogrel”. Citations recalled were initially screened with title and abstract, and then the investigators retrieved and assessed the full texts of potentially relevant studies for their eligibility.

The inclusion criteria were (i) randomized controlled trials (RCTs), published in English; (ii) patients with ACS, with at least two different P2Y12 inhibitors; (iii) older patients with a mean age ≥ 60 years; and (iv) provided information on any of the prespecified primary, secondary and safety endpoints. In the case of more than one publication for one study, the article that met the inclusion criteria and provided sufficient information was included.

### Data collection

Two investigators independently extracted data on study designs, measurements, patient characteristics, and outcomes using a standardized data extraction form. Data collection included authors, year of publication, inclusion and exclusion criteria, sample size, baseline clinical characteristics of patients, observed adverse events, and medical treatment, as available. For studies where intention-to-treat analysis was performed and the results reported, we collected the results of the intention-to-treat analysis for meta-analysis. To improve data extraction, supplementary materials, sub-studies, and pooled analyses pertinent to the study of interest were also examined.

### Risk of bias assessment

Risk of bias was assessed using the Cochrane “Risk of Bias-2” tool (ROB-2) [17]. It evaluates the risk of bias with five domains considered: randomization process, deviations from intended interventions, and missing outcome data. measurement of the outcome and selection of the reported result. This was performed independently for eligible RCTs by two reviewers, with disagreements resolved by involvement of a third reviewer.

### Clinical outcomes

The primary outcome was trial-defined MACE, which was defined as a composite of cardiovascular death, myocardial infarction, and stroke if available, but a composite of death, myocardial infarction and stroke could be an alternative. The secondary outcomes were all-cause mortality, cardiovascular death, myocardial infarction, stroke, stent thrombosis, and trial defined major bleeding. Major bleeding events were defined in the studies variously as Thrombolysis in Myocardial Infarction (TIMI) major bleeding, non-coronary artery bypass grafting major bleeding, PLATelet inhibition and patient Outcomes (PLATO) major bleeding, bleeding requiring transfusion or prologed hospitalization, and Bleeding Academic Research Consortium (BARC) 3 and 5 bleeding. Definitions of endpoints in individual studies are listed in Supplementary Table 2.

### Statistical analysis

A Bayesian multiple treatment network meta-analysis with random effects and uninformative priors was performed. The main analysis was performed on all eligible trials and in the subgroup excluding trials at high risk of bias. Glass Δ was used as the standardized mean difference (SMD) measure, with a 95% credibility interval (CrI). An SMD of 0.20 was considered a small difference between the experimental and the control group; 0.50, a moderate difference; and 0.80, a large difference. Analysis was performed using the Markov-chain Monte Carlo method, based on 100,000 iterations with a burn-in of 50,000.

Homogeneity and consistency assumptions were verified using node splitting and the Bland Altman method. The DerSimonian–Laird random-effects model was used to estimate the variance in heterogeneity between studies. For each iteration, treatments were ranked by their effect relative to an arbitrary baseline. The findings were interpreted as associations when the 95% CrI excluded the null value. A frequency table was constructed from these rankings and normalized by the number of iterations giving the rank probabilities. Convergence was assessed using standard diagnostics.

Probability values were summarized and reported as the surface under the cumulative ranking (SUCRA) curve and with a rankogram plot to provide a hierarchy of treatments with consideration of both the location and the variance of all relative treatment effects. The SUCRA value would be 0 when a treatment is certain to be the worst and 1 when it is certain to be the best. All analyses were conducted using R-evolution version 4.1.2. The meta-analysis was conducted and reported according to the PRISMA (Preferred Reporting Items for Systematic Reviews and Meta-Analyses) statements.

## Results

### Study search and study characteristics

**Fig. 1:**
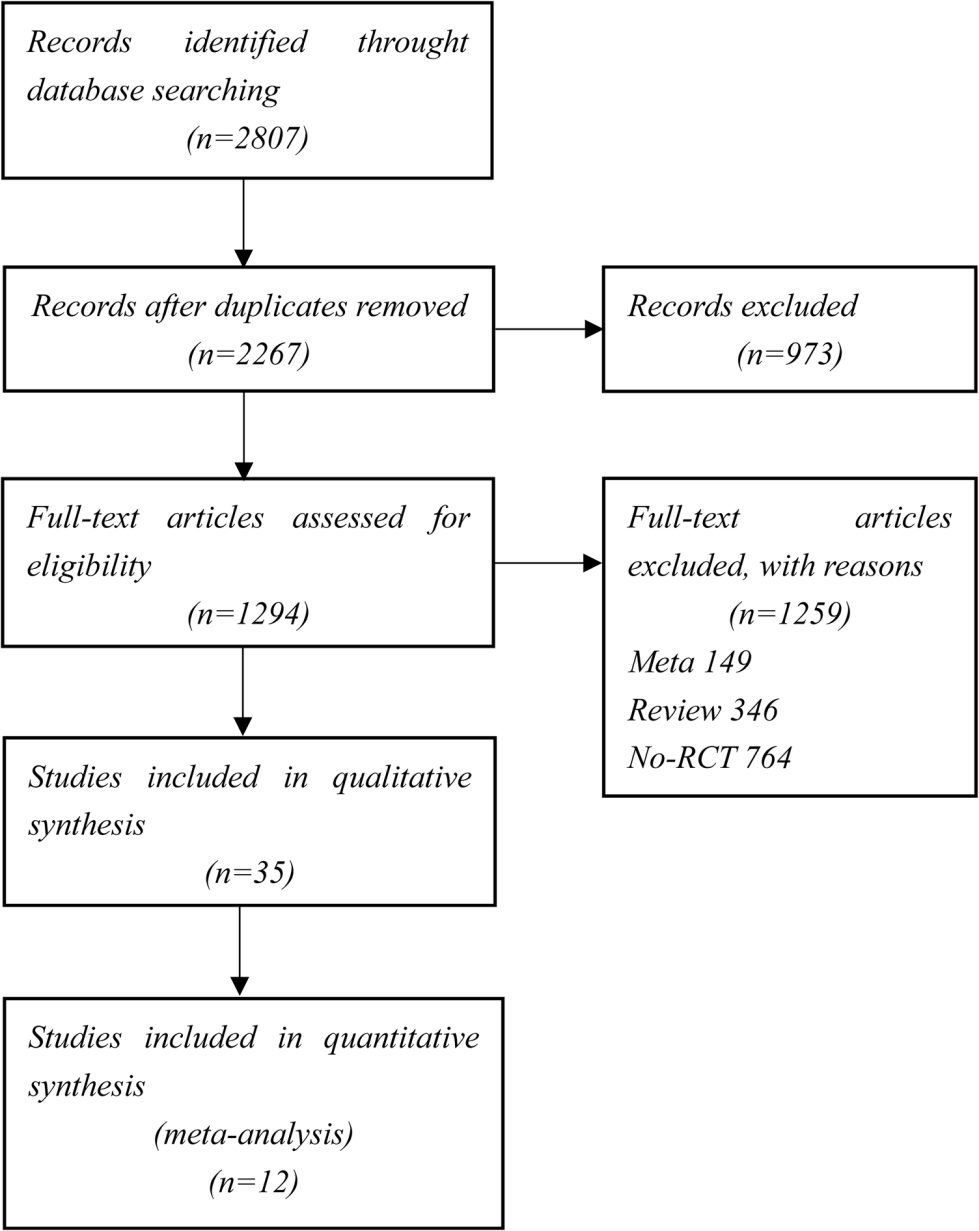
Selection of studies. The preferred reporting items for systematic review and meta-analyses (PRISMA) flow diagram of study selection

After removing duplicates, a total of 2267 potentially relevant articles were screened. Eventually, 12 eligible RCTs were identified, involving a total of 59,284 older patients. These 12 studies were TWILIGHT-ACS [18], TRITON-TIMI 38 [19], PLATO [20], TRILOGY ACS [21], PRASFIT ACS [22], Wang et al. 2016 [23], PRAGUE-18 [24], Elderly ACS II [25], ISAR-REACT5 [26], POPular AGE [27], TICO [28], and TICAKOREA [29]. Regarding anti-platelet therapies strategies, four trials examined prasugrel to clopidogrel, while four trials compared ticagrelor to clopidogrel. Two trials compared prasugrel to ticagrelor. The median follow-up duration of the included studies was 12 months. Six trials reported the results based on intention-to-treat analysis [18–20, 26, 27, 29]. The process of study selection is illustrated in Fig. 1, and detailed information of the included studies is summarized in Table 1. The outcomes of all included and the definitions of MACE and bleeding are list in Supplemental Tables 1 and 2. The risk of bias was assessed by ROB-2 for each RCT and the barplot shown in Supplemental Fig. 1.

**Table 1:** Characteristics of studies included in the systematic review and meta-analysis

	Comparison	Patient number	Age (Years)	Men (%)	Hypertension (%)	Hyperlipidemia (%)	Diabetes Mellitus (%)	Smoking (%)	Previous MI (%)	Previous PCI (%)	Previous CABG (%)	Presentation(%)		
												STEMI	NSTEMI	UA
TRITON-TIMI 38	P(60 mg + 10 mg/d)	6813	60.9(11.2)	75	NA	NA	NA	NA	NA	NA	NA	26	72.9	
	C(300 mg + 75 mg/d)	6795	60.9(11.4)	73.2	NA	NA	NA	NA	NA	NA	NA	27.7	72.3	
PLATO	T(90 mg*bid)	9333	62.1(11.21)	71.6	65.8	46.6	24.9	36	20.4	13.6	5.7	37.5	42.9	16.6
	C(75 mg/d)	9291	62.3(11.21)	71.7	65.1	46.7	25.1	35.7	20.7	13.1	6.2	38	42.5	16.8
TRILOGY ACS	P(30 mg + 5/10 mg/d)	4663	61.4 (8.55); 80.3 (4.29)	60.8	NA	NA	37.7	NA	42.5	34.5	NA	0	70.4	24.2
	C(300 mg + 75 mg/d)	4663	61.5 (8.38); 80.3 (4.39)	60.9	NA	NA	38.3	NA	24.8	35.7	NA	0	69.4	25.1
PRASFIT ACS	P(20 mg + 3.75 mg/d)	685	65.4(11.4)	78.2	72.3	75.3	36.5	NA	5	NA	0.9	49.6	27.3	22.8
	C(300 mg + 75 mg/d)	678	65.1(11.3)	79.4	72.4	73.7	35	NA	5.2	NA	0.6	50.3	31.4	18.3
Wang et al. 2016	C(300 mg + 75 mg/d)	100	80(74,86)	66	82	79	39	41	15	6	0	32	47	21
	T(180 mg + 90 mg*2/d)	100	79(76,85)	69	79	84	42	37	17	3	0	37	44	19
PRAGUE-18	P(30 mg + 10 mg/5 mg/d)	634	61.8(42.7,78.7)	77.1	51.4	33.4	20	64	7.4	6.6	1.9	89.6	5.2	NA
	T(180 mg + 90 mg*2/d)	596	61.8(44.6,79.8)	73.7	51.2	35.4	20.8	65.8	9.2	7.6	1.5	89.4	5.7	NA
Elderly ACS II	P(60 mg + 5 mg/d)	713	80(77,84)	59	78	47	30	9	19	20	8	NA	NA	NA
	C(300 mg + 75 mg/d)	730	80(77,84)	61	78	43	28	9	19	16	10	NA	NA	NA
ISAR-REACT5	T(180 mg + 90 mg*2/d)	2012	64.5(12)	76.2	71.3	58.7	23	34.1	15.5	22.5	5.7	41.4	46.2	12.4
	P(60 mg + 5/10 mg/d)	2006	64.6(12.1)	76.2	69.1	58.1	21.4	33.4	16	23.1	6.5	40.9	46.1	13
POPular AGE	C(300 mg + 75 mg/d)	500	77(73,81)	63	73	65	29	14	24	20	17	0	86	11
	T(180 mg + 90 mg*2/d)	502	77(73,82)	65	73	65	30	13	27	24	17	0	86	11
TWILIGHT-ACS	T 3 m	2273	64.2 (10.5)	74.5	67.5	NA	35.6	23.3	25.4	34.2	8.8	0	100	0
	DAPT 1y	2341	64.2 (10.6)	75.2	67.4	NA	34.3	26.6	25.2	34.4	8.5	0	100	0
TICO	T 3 m	1527	61 (11)	79	50	61	27	36	4	9	1	36	35	29
	DAPT 1y	1529	61 (11)	80	51	60	27	38	3	8	1	36	32	32
TICAKOREA	T(180 mg + 90 mg*2/d)	400	62.5 (11.3)	74.2	55.8	52.0	29	6	6.2	10.2	1	NA	NA	NA
	C(300/600 mg + 75 mg/d)	400	62.3 (11.5)	75.5	48.2	48.5	25	4	5	7.8	0.8	NA	NA	NA

*a Age is median* median (interquartile range), or mean SD, *CABG* coronary artery bypass grafting, *MI* myocardial infarction, *NA* not available, *NSTEMI* non-ST-segment elevation myocardial infarction, *PCI* percutaneous coronary intervention, *STEMI* ST-elevation myocardial infarction, *UA* unstable angina (%)

### Primary outcome: MACE

In total, 5666 (9.56%) patients experienced MACEs, with 1540 (9.93%) of the 15,514 with prasugrel, 1595 (9.42%) of the 16,940 with ticagrelor, and 3036 (12.60%) of the 24,099 treated with clopidogrel.

**Fig. 2:**
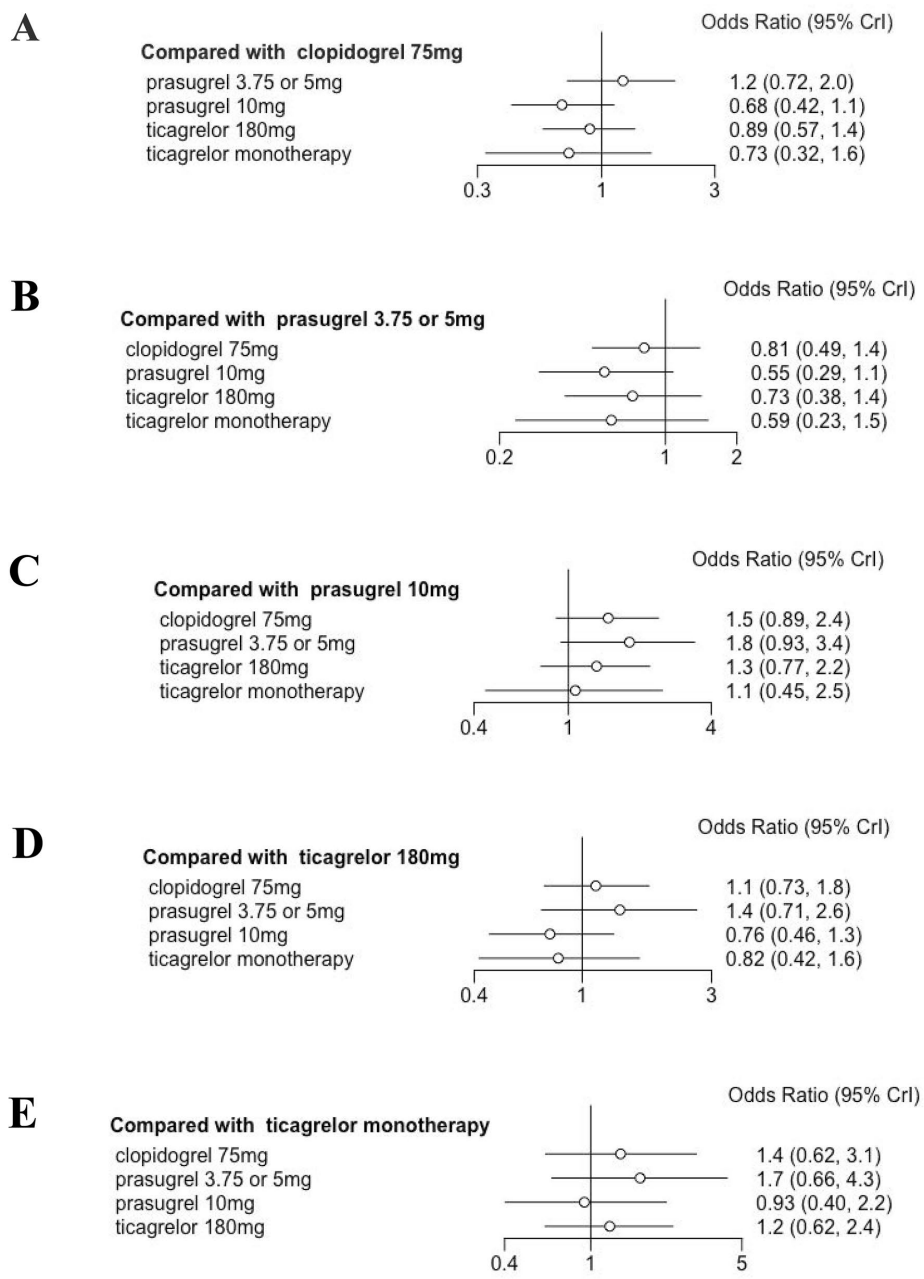
Primary outcome: MACE

**Fig. 3:**
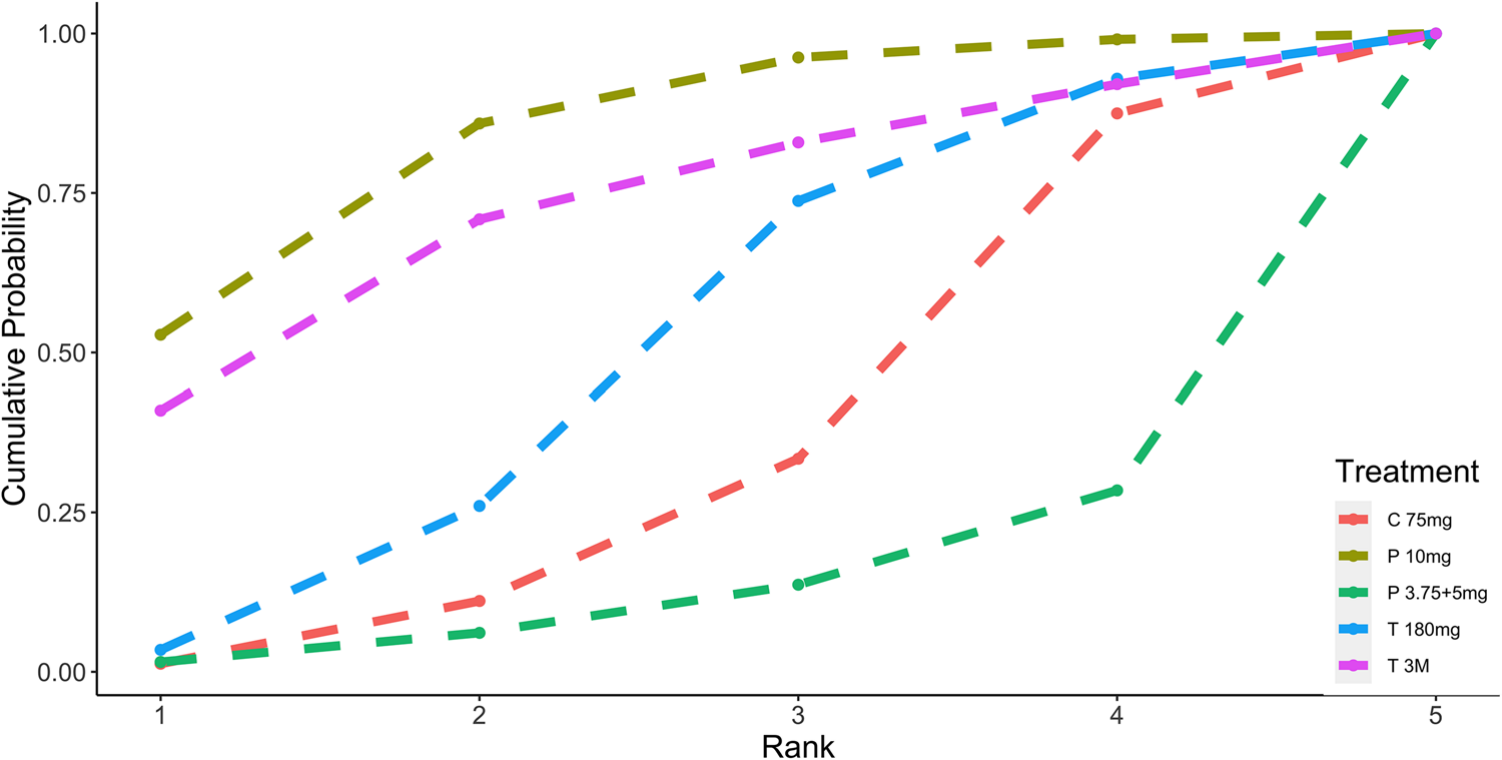
Ranking plots for MACE

Standard meta-analysis of these studies revealed that DAPT of prasugrel 3.75 or 5 mg [1.2 (0.72, 2.0)], prasugrel 10 mg [0.68 (0.42,1.1)], ticagrelor 180 mg [0.89 (0.57, 1.4)] and ticagrelor monotherapy after 3 months DAPT [0.73 (0.32, 1.6)] showed similar primary endpoints when compared to clopidogrel (Fig. 2). The P score indicated that prasugrel 10 mg (0.528) as the most effective treatment for MACE, followed by ticagrelor monotherapy after 3 months DAPT (0.410), ticagrelor 180 mg (0.035) and clopidogrel 75 mg (0.018), while prasugrel 3.75 or 5 mg (0.015) was ranked the lowest (Fig. 3). Nonetheless, SUCRA Bayesian analysis demonstrated that prasugrel 10 mg (0.835) and ticagrelor monotherapy after 3 months DAPT (0.717) were the preferable treatment compared with ticagrelor 180 mg (0.490), clopidogrel 75 mg (0.333) and prasugrel 3.75 or 5 mg (0.124) for the primary endpoints of MACE.

### Secondary outcomes: all-death mortality, CV death, stroke, MI, revascularization and stent thrombosis

**Fig. 4:**
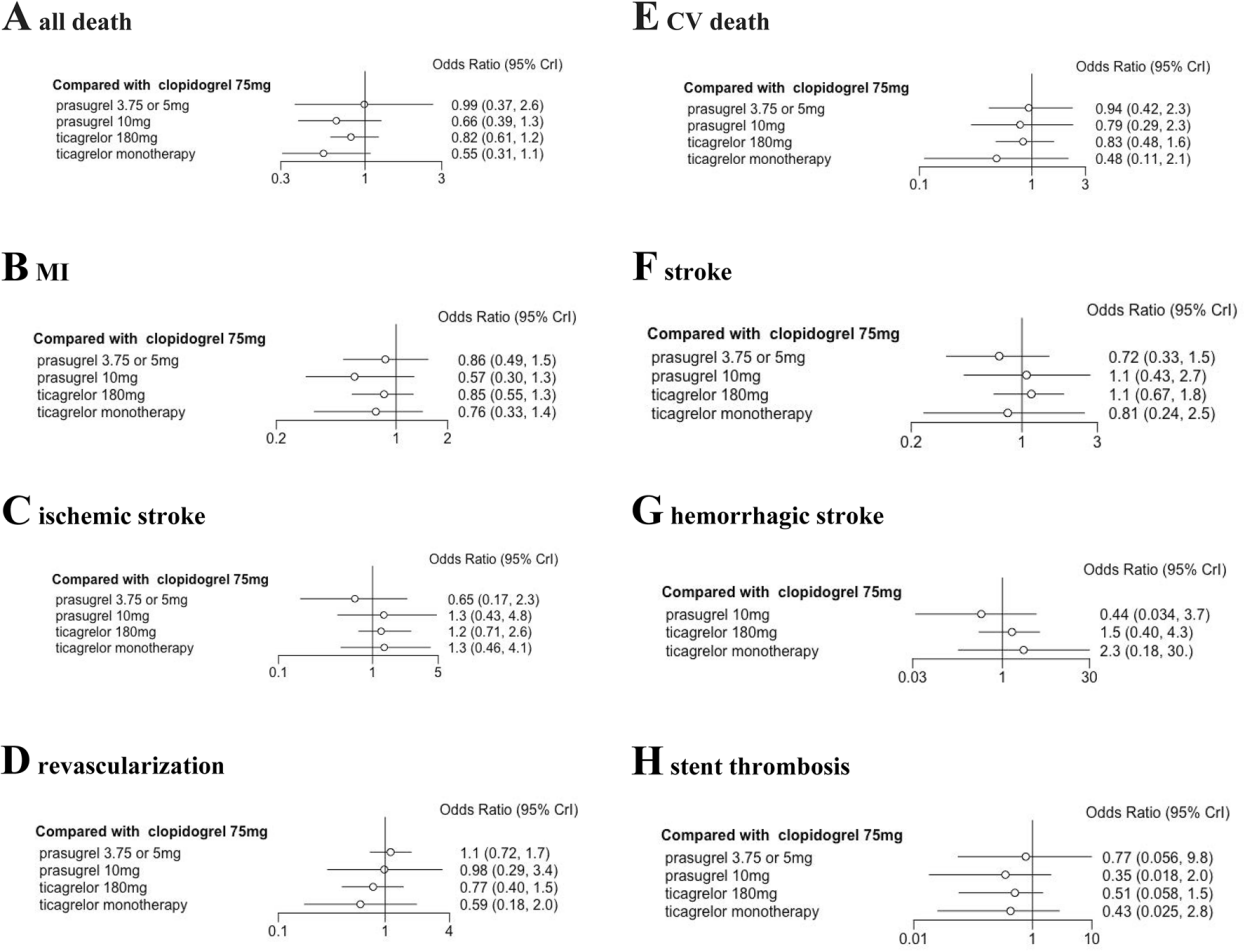
Secondary outcomes compared with clopidogrel 75mg

Compared with DAPT of clopidogrel 75 mg, there was no significant difference in secondary outcomes, such as all-cause death, CV death, stroke, MI, revascularization and stent thrombosis, with prasugrel 3.75 or 5 mg, prasugrel 10 mg, ticagrelor 180 mg or ticagrelor monotherapy after 3 months DAPT, as illustrated in Fig. 4.

**Fig. 5:**
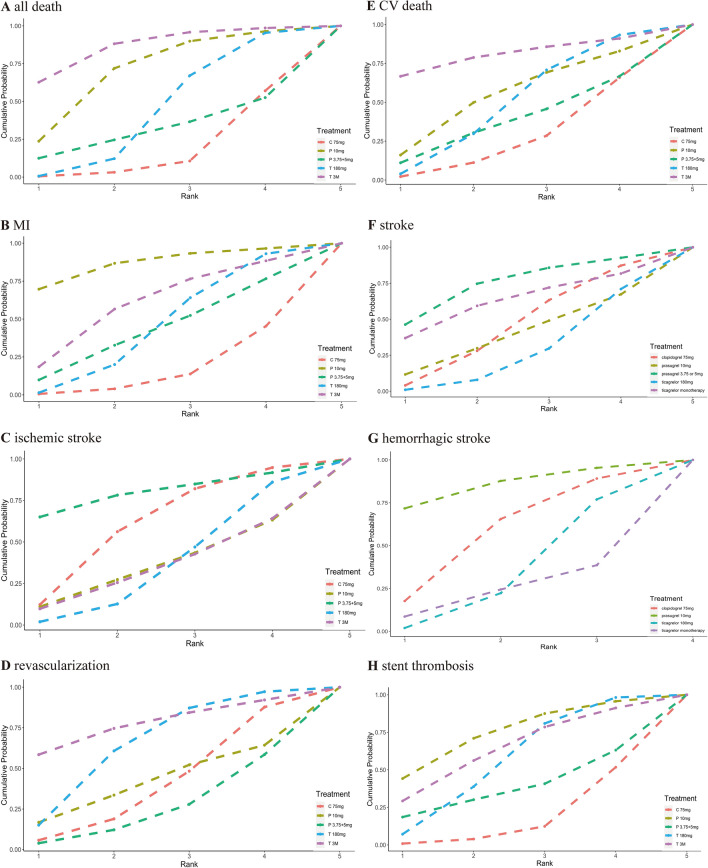
Ranking plots for secondary outcomes

According to P score analysis, ticagrelor monotherapy after 3 months DAPT was the most beneficial treatment for all deaths (0.627), cardiovascular deaths (0.667) and revascularization (0.585). Prasugrel 3.75 or 5 mg was identified as the most effective treatment for stroke (0.463) and ischemic stroke (0.650), while prasugrel 10 mg was most likely the optimal treatment for hemorrhagic stroke (0.717), MI (0.697), and stent thrombosis (0.442), as demonstrated in Fig. 5. However, SUCRA Bayesian analysis revealed that ticagrelor monotherapy after 3 months DAPT was the superior treatment strategy for primary endpoint of all death (0.863), cardiovascular death (0.806) and revascularization (0.774). SUCRA Bayesian analysis also showed that prasugrel 3.75 or 5 mg was the most effective for stroke (0.743) and ischemic stroke (0.800), while prasugrel 10 mg was the ideal treatment in terms of hemorrhagic stroke (0.850), MI (0.866) and stent thrombosis (0.746).

### Bleeding outcome

**Fig. 6:**
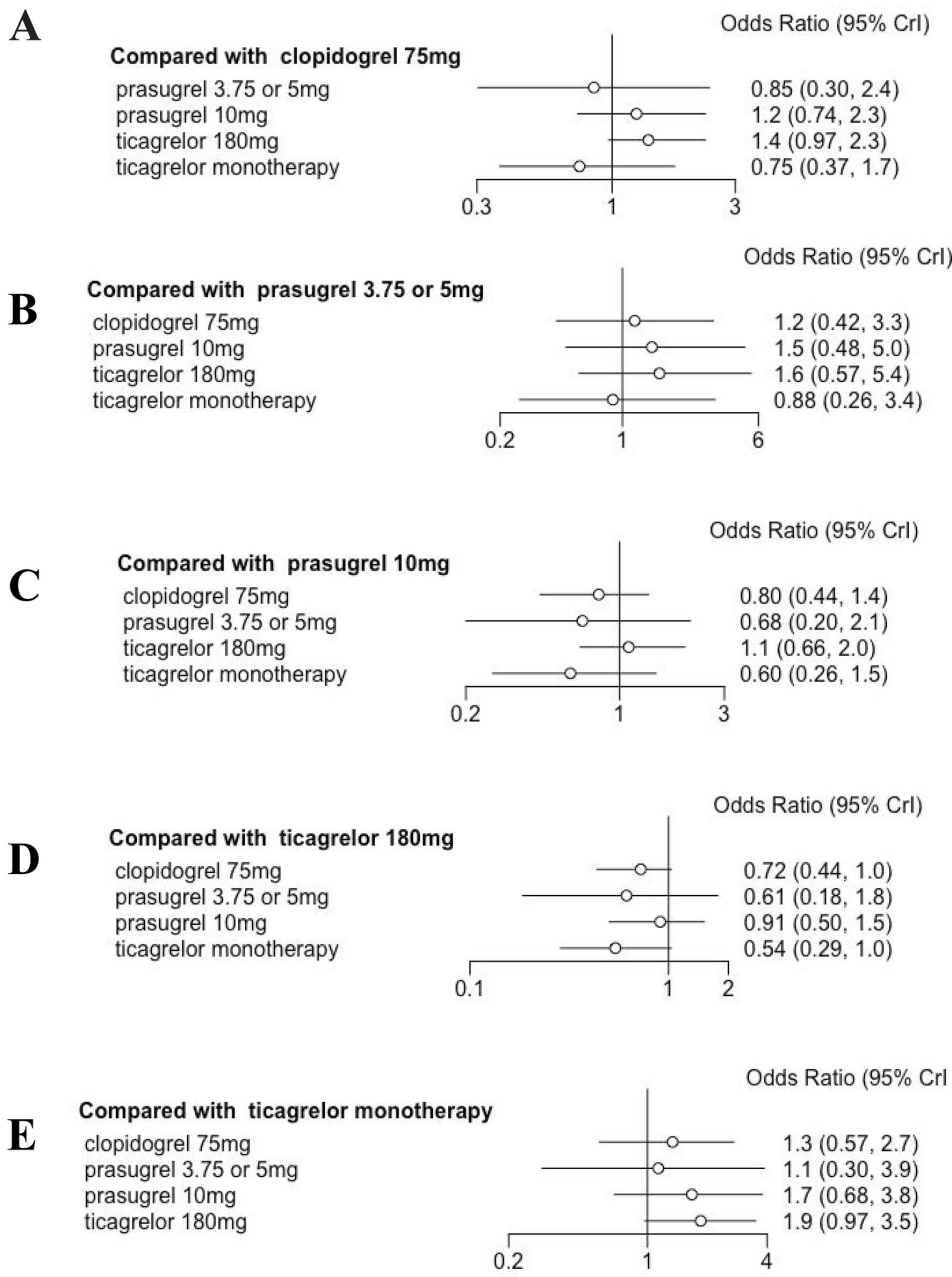
Bleeding outcomes

**Fig. 7:**
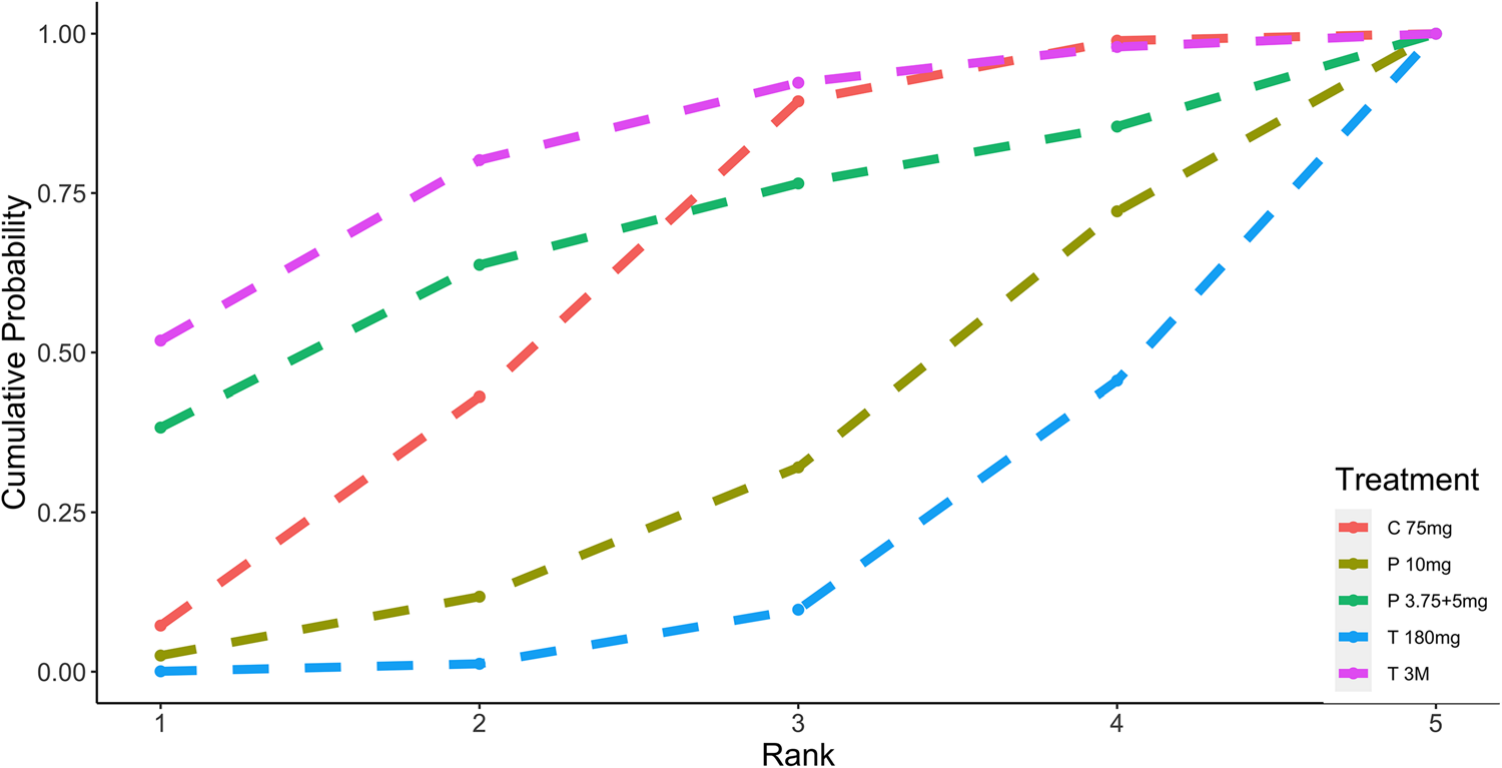
Ranking plots for major bleedings

There was heterogeneity in the definition of bleeding (Supplemental Table 1) among the RCTs that were included, but this standard was deemed acceptable for the purpose of this analysis. Through standard meta-analytic approach, DAPT of prasugrel 3.75 or 5 mg [0.85 (0.30, 2.4)], prasugrel 10 mg [1.2 (0.74, 2.3)], ticagrelor 180 mg [1.4 (0.97, 2.3)], and ticagrelor monotherapy after 3 months DAPT [0.75 (0.37, 1.7)] showed similar primary endpoints in comparison to clopidogrel (as shown in Fig. 6). The P score indicated that ticagrelor monotherapy after 3 months DAPT (0.519) was the optimal treatment for bleeding, followed by prasugrel 10 mg (0.025), prasugrel 3.75 mg (0.383) and clopidogrel 75 mg (0.072), whereas ticagrelor 180 mg (0.0006) was ranked the lowest (as shown in Fig. 7). Nonetheless, SUCRA Bayesian analysis demonstrated that ticagrelor monotherapy after 3 months DAPT (0.806) was the superior treatment concerning bleeding when compared to clopidogrel 75 mg (0.597), prasugrel 3.75 mg (0.660), prasugrel 10 mg (0.296) and ticagrelor 180 mg (0.142).

### Sensitivity analysis and publication bias

In the sensitivity analysis, we used a fixed-effects model to re-analyze the data, and the results remained relatively consistent, indicating robust and trustworthy results (Supplement 6). The funnel plot did not reveal significant publication bias regarding (Supplement Fig. 5).

## Discussion

This systematic review and network meta-analysis comprehensively analyzed the RCTs on the efficacy and safety of different anti-platelet therapies for older patients with ACS. In terms of MACE events, ticagrelor monotherapy after 3 months DAPT was comparable to the other strategies, as well as for all death, CV death and revascularization was considered as the most effective treatment. In addition, ticagrelor monotherapy was the safest option of bleeding risk base on both SUCRA Bayesian and P score rank. The main finding of this study was that ticagrelor monotherapy after 3 months DAPT may be a promising strategy for achieving a more favorable balance between risk and benefit for older ACS patients.

The challenge of therapy in the older patients, particularly anti-platelet therapy in older adults with ACS, was highlighted several decades ago, considering changes in pathophysiology and limited clinical evidence [4, 6]. Aging changes the pharmacokinetics and pharmacodynamics of drugs [30]. Biological changes, including diminished renal clearance, hepatic clearance, muscle mass and greater body fat, lead to altered distribution, metabolism, and elimination of drugs, which increases the risk of ADEs in older adults [31]. Age-related pharmacodynamic changes include altered end-organ responsiveness to drugs and reduced cardiac and baroreflex responses [32]. Older patients have high embolic risk and high bleeding risk, which requires caution [13]. Moreover, the phenotype of atherosclerosis itself may be different, with more extensive coronary calcification and a higher likelihood of multivessel disease [7]. The challenge posed by these characteristics is that evidence based on younger populations may have limited applicability to older patients.

The current network and direct pairwise meta-analysis demonstrate that ticagrelor monotherapy after 3 months DAPT, prasugrel 3.75 or 5 mg and clopidogrel 75 mg are associated with a relative lower risk of bleeding when compared to prasugrel 10 mg and ticagrelor 180 mg. Stronger anti-platelet effect is commonly associated with a higher risk of bleeding, so an appropriate anti-platelet strategy is important for ACS patients, especially for older adults. There are safety concerns regarding the use of standard doses of drugs due to an increased risk of bleeding in older patients. De-escalation of DAPT is now favored to limit adverse outcomes.

A previous meta-analysis [33] of bleeding risk with ticagrelor in older adults over 75 years of age reported that older ACS patients treated with ticagrelor have a 20% increased risk of a bleeding event compared to clopidogrel. Our results showed the highest risk of bleeding in ticagrelor 180mg (SUCRA 0.142). There was a relative higher risk of bleeding compared to clopidogrel [0.75 (0.37 1.7)]. 3 months DAPT followed by ticagrelor monotherapy is the safest in terms of bleeding (0.806) and may be an appropriate strategy for older ACS patients.

For efficacy, our direct pairwise and network meta-analysis demonstrated no significant difference between these strategies. SUCRA Bayesian analysis showed that ticagrelor monotherapy after 3 months DAPT was the best treatment in terms of the primary endpoint of all death, cardiovascular death and revascularization. While prasugrel 3.75 or 5 mg was identified as the most effective for stroke and ischemic stroke, prasugrel 10 mg was most likely the optimal treatment for hemorrhagic stroke, MI and stent thrombosis. In contrast to our findings, another network meta-analysis [15, 34] reported that prasugrel, ticagrelor and clopidogrel showed different efficacies in ischemic events. This may be related to the different age ranges of the population included with different embolic risks, and diverse dosages of P2Y12 inhibitors with confounding factors. On the other hand, similar results were confirmed in another meta-analysis [35] of patients with ACS after PCI. Their quantitative analysis showed treated with P2Y12 inhibitor monotherapy as compared with standard DAPT without increasing the risk of MACE (OR 0.98, 95% CI 0.86–1.13, p = 0.82), and ticagrelor monotherapy is superior to clopidogrel monotherapy.

This study had several limitations. First, we did not have access to the individual patient data to conduct individual patient-level analysis. Second, RCTs included in this meta-analysis differed based on their design, eligibility and exclusion criteria, and some of their study endpoints. However, our analysis did not find substantial heterogeneity between the studies for the endpoint assessed. Despite these limitations, the results of this meta-analysis are important for clinical care and policy.

## Conclusions

The current evidence demonstrated that ticagrelor monotherapy after 3 months DAPT may be a promising approach for achieving a more favorable balance between risk and benefit for older ACS patients, with a relatively low bleeding risk and without an increased risk of MACE events. Moreover, it remains the preferred option for clinical outcomes such as all death, CV death and revascularization. Further high-quality and long-term studies are required to validate anti-platelet therapies among older ACS patients.

### Supplementary Information

Below is the link to the electronic supplementary material.

Supplementary file1 (DOCX 126138 kb)
