# Supplementary material for: Optimal anti-platelet therapy for older patients with acute coronary syndrome: a network meta-analysis of randomized trials comprising 59,284 older patients
Source: J Thromb Thrombolysis. 2023 Aug 7;57(1):143–54. doi: 10.1007/s11239-023-02875-x (PMC10830599; doi:10.1007/s11239-023-02875-x)
Supplement: Supplementary file 1 — Supplementary file1 (DOCX 126138 kb) [file 11239_2023_2875_MOESM1_ESM.docx]

**Supplementary Material**

[Part 1 Table	3](#_Toc138325968)

[Supplemental Table 1: MACE and major bleeding outcome definitions	3](#_Toc138325969)

[Supplemental Table 2: Outcomes of all studies	5](#_Toc138325970)

[Part 2: Figure	7](#_Toc138325971)

[1. Risk of bias of the trials included in the meta-analysis.	7](#_Toc138325972)

[2. Network meta-analysis (random effects model).	9](#_Toc138325973)

[Supplemental Figure 2.1: all-death mortality	9](#_Toc138325974)

[Supplemental Figure 2.2: cardiovascular death mortality	11](#_Toc138325975)

[Supplemental Figure 2.3: stroke	13](#_Toc138325976)

[Supplemental Figure 2.4: myocardial infarction	15](#_Toc138325977)

[Supplemental Figure 2.5: revascularization	17](#_Toc138325978)

[Supplemental Figure 2.6: stent thrombosis	19](#_Toc138325979)

[3 Subgroup analysis	21](#_Toc138325980)

[Supplemental Figure 3.1: MACE	21](#_Toc138325981)

[Supplemental Figure 3.2: major bleeding	22](#_Toc138325982)

[4 Analysis of heterogeneity	23](#_Toc138325983)

[Supplemental Figure 4.1: MACE	23](#_Toc138325984)

[Supplemental Figure 4.2: major bleeding	24](#_Toc138325985)

[Supplemental Figure 4.3: all death	25](#_Toc138325986)

[Supplemental Figure 4.4: cardiovascular death mortality	26](#_Toc138325987)

[Supplemental Figure 4.5: stroke	27](#_Toc138325988)

[Supplemental Figure 4.6: myocardial infarction	28](#_Toc138325989)

[Supplemental Figure 4.7: revascularization	29](#_Toc138325990)

[Supplemental Figure 4.8: stent thrombosis	30](#_Toc138325991)

[5 Funnel Plot	3](#_Toc138326001)1

[Part 3: Sensitivity analysis	32](#_Toc138325971)

# Part 1 Table

## Supplemental Table 1: MACE and major bleeding outcome definitions

|  | MACE | Major bleeding |
| --- | --- | --- |
| TRITON-TIMI 38 | Cardiovascular death, nonfatal myocardial infarction, or nonfatal stroke. | TIMI major bleeding |
| PLATO | Cardiovascular death, myocardial infarction or stroke | Non-CABG major bleeding (PLATO defined) |
| TRILOGY ACS | Cardiovascular death, nonfatal myocardial infarction, or nonfatal stroke | TIMI major bleeding |
| PRASFIT ACS | Cardiovascular death, nonfatal myocardial infarction, and nonfatal ischemic stroke. | Non-CABG-related TIMI major bleeding |
| Wang et al. 2016 | Cardiovascular death, myocardial infarction or stroke | PLATO major bleeding |
| PRAGUE-18 | Cardiovascular death,  nonfatal myocardial infarction, or stroke | Bleeding requiring transfusion or prolonging hospitalization |
| Elderly ACS II | NA | BARC 3 and 5 bleeding |
| ISAR-REACT5 | Death, myocardial infarction, or stroke | BARC 3 and 5 bleeding |
| POPular AGE | Cardiovascular death, myocardial infarction or stroke | BARC 3 and 5 bleeding |
| TWILIGHT-ACS | Cardiovascular death, MI or ischemic stroke | TIMI major bleeding |
| TICO | Death, myocardial infarction, stent thrombosis, stroke, or target-vessel revascularization | TIMI major bleeding |
| TICAKOREA | Cardiovascular death, nonfatal MI, or nonfatal stroke | PLATO major bleeding |

NA = not available, MACE = major adverse cardiovascular events

## Supplemental Table 2: Outcomes of all studies

|  | Comparison | Patient number | All Death | Cardiovascular death | Stroke | ischemic stroke | hemorrhagic stroke | revascularization | stent thrombosis | MACE | major bleeding |
| --- | --- | --- | --- | --- | --- | --- | --- | --- | --- | --- | --- |
| TRITON-TIMI 38 | P(60mg+10mg/d) | 6813 | NA | NA | NA | NA | NA | NA | NA | 643 | 146 |
|  | C(300mg+75mg/d) | 6795 | NA | NA | NA | NA | NA | NA | NA | 781 | 111 |
| PLATO | T(90mg*bid) | 9333 | 399 | 353 | 125 | 96 | 23 | NA | 155 | 864 | 657 |
|  | C(75mg/d) | 9291 | 506 | 442 | 106 | 91 | 13 | NA | 202 | 1014 | 638 |
| TRILOGY ACS | P(30mg+10mg/d) | 3620 | NA | NA | NA | NA | NA | NA | NA | 364 | NA |
|  | P(30mg+5mg/d) | 1043 | NA | NA | NA | NA | NA | NA | NA | 257 | NA |
|  | C(300mg+75mg/d) | 3623 | NA | NA | NA | NA | NA | NA | NA | 397 | NA |
|  | C(300mg+75mg/d) | 1040 | NA | NA | NA | NA | NA | NA | NA | 251 | NA |
| PRASFIT ACS | P(20mg+3.75mg/d) | 685 | 11 | 10 | 6 | 6 | NA | 107 | 4 | 74 | 13 |
|  | C(300mg+75mg/d) | 678 | 11 | 8 | 10 | 9 | NA | 96 | 5 | 84 | 15 |
| Wang et al. 2016 | C(300mg+75mg/d) | 100 | 16 | 15 | 3 | NA | NA | NA | NA | 22 | 6 |
|  | T(180mg+90mg*2/d) | 100 | 9 | 6 | 2 | NA | NA | NA | NA | 11 | 8 |
| PRAGUE-18 | P(30mg+10mg/5mg/d) | 634 | 14 | 8 | 2 | NA | NA | 9 | 3 | 17 | 3 |
|  | T(180mg+90mg*2/d) | 596 | 16 | 8 | 1 | NA | NA | 7 | 5 | 15 | 3 |
| Elderly ACS II | P(60mg+5mg/d) | 713 | NA | 25 | 10 | NA | NA | NA | NA | 48 | NA |
|  | C(300mg+75mg/d) | 730 | NA | 31 | 12 | NA | NA | NA | NA | 61 | NA |
| ISAR-REACT5 | T(180mg+90mg*2/d) | 2012 | 90 | 63 | 22 | 16 | 6 | NA | 26 | 184 | 95 |
|  | P(60mg+5/10mg/d) | 2006 | 73 | 59 | 19 | 17 | 2 | NA | 20 | 137 | 80 |
| POPular AGE | C(300mg+75mg/d) | 500 | 37 | 18 | NA | 5 | NA | 12 | 5 | 53 | 9 |
|  | T(180mg+90mg*2/d) | 502 | 34 | 15 | NA | 10 | NA | 9 | 0 | 57 | 21 |
| TWILIGHT-ACS | T 3m | 2273 | 22 | NA | NA | 11 | NA | NA | 8 | 90 | 12 |
|  | DAPT 1y | 2341 | 34 | NA | NA | 6 | NA | NA | 14 | 97 | 23 |
| TICO | T 3m | 1527 | 16 | 7 | 8 | 5 | 3 | 8 | 6 | 35 | 25 |
|  | DAPT 1y | 1529 | 23 | 12 | 11 | 9 | 2 | 10 | 4 | 51 | 45 |
| TICAKOREA | T(180mg+90mg*2/d) | 400 | 16 | 15 | 6 | 5 | 1 | 10 | 2 | 36 | 29 |
|  | C(300/600mg+75mg/d) | 400 | 10 | 6 | 5 | 3 | 2 | 12 | 4 | 23 | 16 |

C = clopidogrel, MACE = major adverse cardiovascular events, NA = Not available, P = prasugrel, T = ticagrelor.

# Part 2: Figure

## 1 Risk of bias of the trials included in the meta-analysis.

Supplemental Figure 1.1 Risk of bias summary

Supplemental Figure 1.2 Risk of bias graph.

## 2 Network meta-analysis (random effects model).

The figure presents odds ratio (OR) and 95% confidence interval (CI) for each treatment arm. (a) network of P2Y12 inhibitor regimen, (b) others versus. Clopidogrel 75mg, (c) others versus. prasugrel 3.75 or 5mg, (d) others versus. prasugrel 10mg, (e) others versus. ticagrelor 180mg, (f) others versus. ticagrelor monotherapy.

## Supplemental Figure 2.1: all-death mortality

(a)

(b)

(c)

(d)

(e)

(f)

## Supplemental Figure 2.2: cardiovascular death mortality

(a)

(b)

(c)

(d)

(e)

(f)

## Supplemental Figure 2.3: stroke

(a)

(b)

(c)

(d)

(e)

(f)

## Supplemental Figure 2.4: myocardial infarction

(a)

(b)

(c)

(d)

(e)

(f)

## Supplemental Figure 2.5: revascularization

(a)

(b)

(c)

(d)

(e)

(f)

## Supplemental Figure 2.6: stent thrombosis

(a)

(b)

(c)

(d)

(e)

(f)

## 3 Subgroup analysis

Subgroup analysis was performed for patients over 70 years of age. (a) others versus. Clopidogrel 75mg, (b) surface under the cumulative ranking (SUCRA) curve.

## Supplemental Figure 3.1: MACE

(a)

(b)

## Supplemental Figure 3.2: major bleeding

(a)

(b)

## 4 Analysis of heterogeneity

| 1 | clopidogrel 75mg |
| --- | --- |
| 2 | prasugrel 3.75 or 5mg |
| 3 | prasugrel 10mg |
| 4 | ticagrelor 180mg |
| 5 | ticagrelor monotherapy |

## Supplemental Figure 4.1: MACE

## Supplemental Figure 4.2: major bleeding

## Supplemental Figure 4.3: all death

## Supplemental Figure 4.4: cardiovascular death mortality

## Supplemental Figure 4.5: stroke

## Supplemental Figure 4.6: myocardial infarction

## Supplemental Figure 4.7: revascularization

## Supplemental Figure 4.8: stent thrombosis

## 5 Funnel Plot

## Supplemental Figure 5.1: MACE

P = 0.9306 (Egger)

## Supplemental Figure 5.2: major bleeding

P = 0.9402 (Egger)

## 6 Sensitivity analysis

Re-analyze (fixed effect model)

## 6.1 MACE

Results on the Log Odds Ratio scale

Iterations = 10001:60000

Thinning interval = 1

Number of chains = 4

Sample size per chain = 50000

1. Empirical mean and standard deviation for each variable,

plus standard error of the mean:

Mean	SD Naive SE Time-series SE

d.1.2 0.4669 0.07118 1.592e-04	0.0003051

d.1.3 -0.3223 0.04078 9.118e-05	0.0001870

d.1.4 -0.1417 0.04327 9.676e-05	0.0002073

d.4.5 -0.1545 0.12286 2.747e-04	0.0005707

2. Quantiles for each variable:

2.5%	25%	50%	75%	97.5%

d.1.2 0.3268 0.4190 0.4670 0.51512 0.60588

d.1.3 -0.4023 -0.3497 -0.3221 -0.29481 -0.24253

d.1.4 -0.2267 -0.1707 -0.1418 -0.11259 -0.05702

d.4.5 -0.3957 -0.2372 -0.1544 -0.07136 0.08570

-- Model fit (residual deviance):

Dbar	pD	DIC

69.20245 16.15075 85.35321

25 data points, ratio 2.768, I^2 = 65%

## 6.2 major bleeding

Results on the Log Odds Ratio scale

Iterations = 10001:60000

Thinning interval = 1

Number of chains = 4

Sample size per chain = 50000

1. Empirical mean and standard deviation for each variable,

plus standard error of the mean:

Mean	SD Naive SE Time-series SE

d.1.2 -0.1603 0.38839 0.0008685	0.0018336

d.1.3 0.1313 0.09960 0.0002227	0.0005171

d.1.4 0.0942 0.05365 0.0001200	0.0002777

d.4.5 -0.6148 0.20731 0.0004636	0.0008443

2. Quantiles for each variable:

2.5%	25%	50%	75%	97.5%

d.1.2 -0.92925 -0.41990 -0.15812 0.1003 0.5937

d.1.3 -0.06442 0.06448 0.13137 0.1989 0.3261

d.1.4 -0.01163 0.05807 0.09432 0.1303 0.1990

d.4.5 -1.02657 -0.75287 -0.61227 -0.4742 -0.2152

-- Model fit (residual deviance):

Dbar	pD	DIC

25.72490 14.08249 39.80738

20 data points, ratio 1.286, I^2 = 26%

## 6.3 all-death mortality

Results on the Log Odds Ratio scale

Iterations = 10001:60000

Thinning interval = 1

Number of chains = 4

Sample size per chain = 50000

1. Empirical mean and standard deviation for each variable,

plus standard error of the mean:

Mean	SD Naive SE Time-series SE

d.1.2 -0.01204 0.43760 0.0009785	0.0021583

d.1.4 -0.23343 0.06445 0.0001441	0.0002939

d.4.3 -0.21439 0.14867 0.0003324	0.0007839

d.4.5 -0.39755 0.21069 0.0004711	0.0009060

2. Quantiles for each variable:

2.5%	25%	50%	75%	97.5%

d.1.2 -0.8728 -0.3048 -0.01174 0.2807 0.84713

d.1.4 -0.3602 -0.2767 -0.23331 -0.1900 -0.10754

d.4.3 -0.5060 -0.3142 -0.21434 -0.1144 0.07680

d.4.5 -0.8172 -0.5380 -0.39637 -0.2553 0.01183

-- Model fit (residual deviance):

Dbar	pD	DIC

17.56943 13.03227 30.60171

18 data points, ratio 0.9761, I^2 = 3%

## 6.4 cardiovascular death mortality

Results on the Log Odds Ratio scale

Iterations = 10001:60000

Thinning interval = 1

Number of chains = 4

Sample size per chain = 50000

1. Empirical mean and standard deviation for each variable,

plus standard error of the mean:

Mean	SD Naive SE Time-series SE

d.1.2 -0.09808 0.23833 0.0005329	0.0011274

d.1.4 -0.22623 0.06995 0.0001564	0.0003230

d.4.3 -0.06562 0.17281 0.0003864	0.0008579

d.4.5 -0.57031 0.49264 0.0011016	0.0020929

2. Quantiles for each variable:

2.5%	25%	50%	75%	97.5%

d.1.2 -0.5663 -0.2585 -0.09751 0.06254 0.36668

d.1.4 -0.3638 -0.2734 -0.22639 -0.17895 -0.08928

d.4.3 -0.4046 -0.1820 -0.06542 0.05086 0.27374

d.4.5 -1.5718 -0.8930 -0.56135 -0.23687 0.37249

-- Model fit (residual deviance):

Dbar	pD	DIC

22.74441 13.09691 35.84132

18 data points, ratio 1.264, I^2 = 25%

## 6.5 stroke

Results on the Log Odds Ratio scale

Iterations = 10001:60000

Thinning interval = 1

Number of chains = 4

Sample size per chain = 50000

1. Empirical mean and standard deviation for each variable,

plus standard error of the mean:

Mean	SD Naive SE Time-series SE

d.1.2 -0.32894 0.3351 0.0007492	0.0014937

d.1.4 0.14994 0.1287 0.0002879	0.0006649

d.4.3 -0.08685 0.3058 0.0006838	0.0015458

d.4.5 -0.34446 0.4802 0.0010738	0.0021516

2. Quantiles for each variable:

2.5%	25%	50%	75% 97.5%

d.1.2 -0.9941 -0.55137 -0.32487 -0.1028 0.3200

d.1.4 -0.1021 0.06273 0.14970 0.2365 0.4037

d.4.3 -0.6904 -0.29157 -0.08565 0.1192 0.5096

d.4.5 -1.3070 -0.66267 -0.33657 -0.0215 0.5815

-- Model fit (residual deviance):

Dbar	pD	DIC

13.20064 12.10149 25.30213

16 data points, ratio 0.825, I^2 = 0%

## 6.6 myocardial infarction

Results on the Log Odds Ratio scale

Iterations = 10001:60000

Thinning interval = 1

Number of chains = 4

Sample size per chain = 50000

1. Empirical mean and standard deviation for each variable,

plus standard error of the mean:

Mean	SD Naive SE Time-series SE

d.1.2 -0.15449 0.16528 0.0003696	0.0007705

d.1.4 -0.16817 0.05899 0.0001319	0.0002766

d.4.3 -0.43589 0.15981 0.0003573	0.0009030

d.4.5 -0.06319 0.16222 0.0003627	0.0007780

2. Quantiles for each variable:

2.5%	25%	50%	75%	97.5%

d.1.2 -0.4791 -0.2656 -0.15494 -0.04347 0.17025

d.1.4 -0.2839 -0.2080 -0.16807 -0.12821 -0.05283

d.4.3 -0.7512 -0.5423 -0.43559 -0.32774 -0.12414

d.4.5 -0.3820 -0.1726 -0.06264 0.04660 0.25190

-- Model fit (residual deviance):

Dbar	pD	DIC

21.48785 14.04493 35.53277

20 data points, ratio 1.074, I^2 = 12%

## 6.7 revascularization

Results on the Log Odds Ratio scale

Iterations = 10001:60000

Thinning interval = 1

Number of chains = 4

Sample size per chain = 50000

1. Empirical mean and standard deviation for each variable,

plus standard error of the mean:

Mean	SD Naive SE Time-series SE

d.1.2 0.1130 0.1524 0.0003409	0.0007701

d.1.4 -0.2638 0.3117 0.0006970	0.0014154

d.4.3 0.2323 0.5153 0.0011523	0.0023574

d.4.5 -0.2605 0.4835 0.0010812	0.0022134

2. Quantiles for each variable:

2.5%	25%	50%	75% 97.5%

d.1.2 -0.1868 0.01081 0.1127 0.21566 0.4122

d.1.4 -0.8799 -0.47187 -0.2625 -0.05309 0.3416

d.4.3 -0.7656 -0.11491 0.2253 0.57090 1.2672

d.4.5 -1.2259 -0.57973 -0.2550 0.06531 0.6771

-- Model fit (residual deviance):

Dbar	pD	DIC

9.025950 8.975209 18.001159

10 data points, ratio 0.9026, I^2 = 0.3%

## 6.8 stent thrombosis

Results on the Log Odds Ratio scale

Iterations = 10001:60000

Thinning interval = 1

Number of chains = 4

Sample size per chain = 50000

1. Empirical mean and standard deviation for each variable,

plus standard error of the mean:

Mean	SD Naive SE Time-series SE

d.1.2 -0.2640 0.7108 0.0015894	0.003357

d.1.4 -0.3034 0.1070 0.0002393	0.000479

d.4.3 -0.3146 0.2797 0.0006255	0.001528

d.4.5 -0.2386 0.3616 0.0008086	0.001664

2. Quantiles for each variable:

2.5%	25%	50%	75%	97.5%

d.1.2 -1.6944 -0.7283 -0.2545 0.210916 1.10699

d.1.4 -0.5138 -0.3755 -0.3029 -0.231414 -0.09432

d.4.3 -0.8673 -0.5022 -0.3128 -0.125728 0.22784

d.4.5 -0.9558 -0.4794 -0.2366 0.004388 0.46620

-- Model fit (residual deviance):

Dbar	pD	DIC

19.70187 12.19491 31.89678

16 data points, ratio 1.231, I^2 = 24%
